# Establishment of a medical device adverse event management system for hospitals

**DOI:** 10.1186/s12913-022-08830-5

**Published:** 2022-11-24

**Authors:** Jing Sun, Jin Pan, Yile Jin, Qian Zhang, Yingying Lv, Jingyi Feng

**Affiliations:** 1 Key Laboratory of Clinical Evaluation Technology for Medical Device of Zhejiang Province, The First Affiliated Hospital, Zhejiang University School of Medicine, Hangzhou, People’s Republic of China; 2 Department of Clinical Engineering, The First Affiliated Hospital, Zhejiang University School of Medicine, Hangzhou, People’s Republic of China

**Keywords:** Management, Medical device adverse event, Continuous quality improvement, Health policy, Risk management

## Abstract

**Background:**

The management of medical device adverse event (MDAE) is one of the most important aspects of improving medical quality and safety management. Nonetheless, hospitals still lack standardized and unified initiatives to improve MDAE management.

**Methods:**

This study, thus, established a MDAE monitoring system on May 1 in 2011 for suspected adverse events and designed a hospital-based dynamic warning system, aiming to standardize the process of MDAE handling and provide real-time monitoring for MDAEs in a hospital. This system was used in the First Affiliated Hospital of Zhejiang University School of Medicine. Numbers and the compound growth rate of MDAE reports from 2010 to 2020 were compared to test the effectiveness of the MDAE monitoring system. Numbers of MDAE reported to the *National Adverse Event Monitoring System* were also compared over 2013 to 2020, due to the loss of data before 2013 after shutdown of the old system. Efficacy and usability of the hospital-based dynamic warning system was then verified by analyzing risk and warning levels of MDAEs in 2020. Descriptive statistics was used for data analysis in this study.

**Results:**

Results showed that the compound annual growth rates of MDAE reports and those submitted to the National Adverse Event Monitoring System from 2013 to 2020 were 35.0% and 31.5%, respectively. A standardized management of MDAE with full participant, timely response and effective feedback was formed in the hospital by establishment of the MDAE system.

**Conclusions:**

This system effectively improved the monitoring level of MDAEs, helping to improve early detection, early warning, and early intervention of risk of medical device. This study may provide suggestions for medical institutions to establish a MDAE monitoring system, and may promote development of medical quality and safety management for hospitals to some extent.

## Introduction

Patient safety is one of the most crucial considerations while developing a medical product in the twenty-first century [1, 2]. With the widespread use of medical devices, risk management becomes more crucial for ensuring the health and safety of patients [3, 4]. To varying degrees, nations pay active attention to adverse events associated with medical technologies. In 1984, the United States established the first system for monitoring and reporting adverse events related to medical device [5]. In 1992, the first Global Harmonization Task Force meeting was held between medical device industry representatives and government agencies from the United States, the European Community, Japan, Canada, and Australia to coordinate medical device management [6]. In 1993, the Food and Drug Administration (FDA) of the United States established the adverse event reporting program, MedWatch, for reporting medical devices use errors [7]. In the aforementioned nations or regions, adverse event management for medical devices has taken the lead in its evolution during the past three decades.

Medical device adverse events (MDAEs) monitoring began late in China, resulting in a monitoring gap compared to the majority of the developed countries or regions. At the beginning of the twenty-first century, the State Food and Drug Administration began monitoring the adverse events of medical devices [8–10], and established a monitoring framework of MDAE. At the same time, a re-evaluation and elimination system for medical devices was established. In December 2008, the State Food and Drug Administration and the Ministry of Health of the People's Republic of China issued the *Administrative Measures for Monitoring and Re-evaluation of Adverse Events of Medical Devices* (for Trial Implementation) [11]. As a result, MDAE monitoring concepts spread through the country. Until August 2018, the State Administration for Market Regulation and the National Health Commission of the People’s Republic of China issued the *Administrative Measures for Monitoring and Re-evaluation of Adverse Events of Medical Devices* (Order No.1 of the State Administration for Market Supervision), which became effective on January 1, 2019 [12]. This regulation defines adverse events of medical as events that may cause injury to humans during normal use of the listed medical devices. Over the last two decades, China’s MDAE monitoring system has been largely successful. Currently, the electronic monitoring platform is utilized extensively throughout China. Over the past decade, the internal monitoring and management system of medical institutions at all levels has been progressively improved. Medical institution staff has become more aware of the need to report adverse events related to medical devices [13].

Enhance medical quality and safety management is an essential objective for medical institutions. Medical quality and safety management has become increasing challenging with the introduction of new and advanced medical devices into clinical practice. Despite the fact that medical institutions have improved the level of MDAE monitoring and management, as well as the reporting awareness of medical personnel, MDAE management currently faces many challenges and problems, such as low efficiency and underutilization historical data. According to Desveaux and Gagliardi, the Theoretical Domains [14] and the Tailored Implementation for Chronic Diseases [15] framework have proven useful in identifying the factors that influence MDAE reporting [16]. The FDA launched the adverse event problem codes to standardize the classification of MDAEs [17]. Medical institutions are actively looking for an efficient method to achieve early detection, early warning and early intervention for MDAE. However, several challenges still need to be addressed to improve the efficiency of MDAE management, the timeliness of MDAE response, and the efficiency of multiparty coordination among medical institutions, dealers, and manufacturers of medical devices. An early warning system for MDAE is essential for medical institutions, particularly for large hospitals. Further, since MDAE reports (MDRs) are generated by medical professionals such as nurses, physicians and clinical engineers, it is essential to make use of them.

The First Affiliated Hospital of Zhejiang University School of Medicine (FAHZU), which has six campuses with more than 5,000 beds and about 9000 employees, manages approximately 31,000 medical devices valued over 2.7 billion RMB. MDAE monitoring is an important part of medical device life cycle management in the FAHZU. The FAHZU was among the first medical facilities in China to use a hospital MDAE monitoring system. In response to the aforementioned problems and obstacles, this study established a MDAE monitoring system for suspected adverse events and improved it by establishing a hospital-based dynamic warning system for MDAE management for hospitals. The MDAE systems and the hospital-based dynamic warning system are evaluated by analyzing MDAE reports in the FAHZU. This study may offer medical institutions a method for establishing an MDAE monitoring system or enhancing an existing one in terms of early detection, early warning and early intervention of medical device risk.

## Methodology

### Design of the workflow of medical device adverse event management

**Fig. 1:**
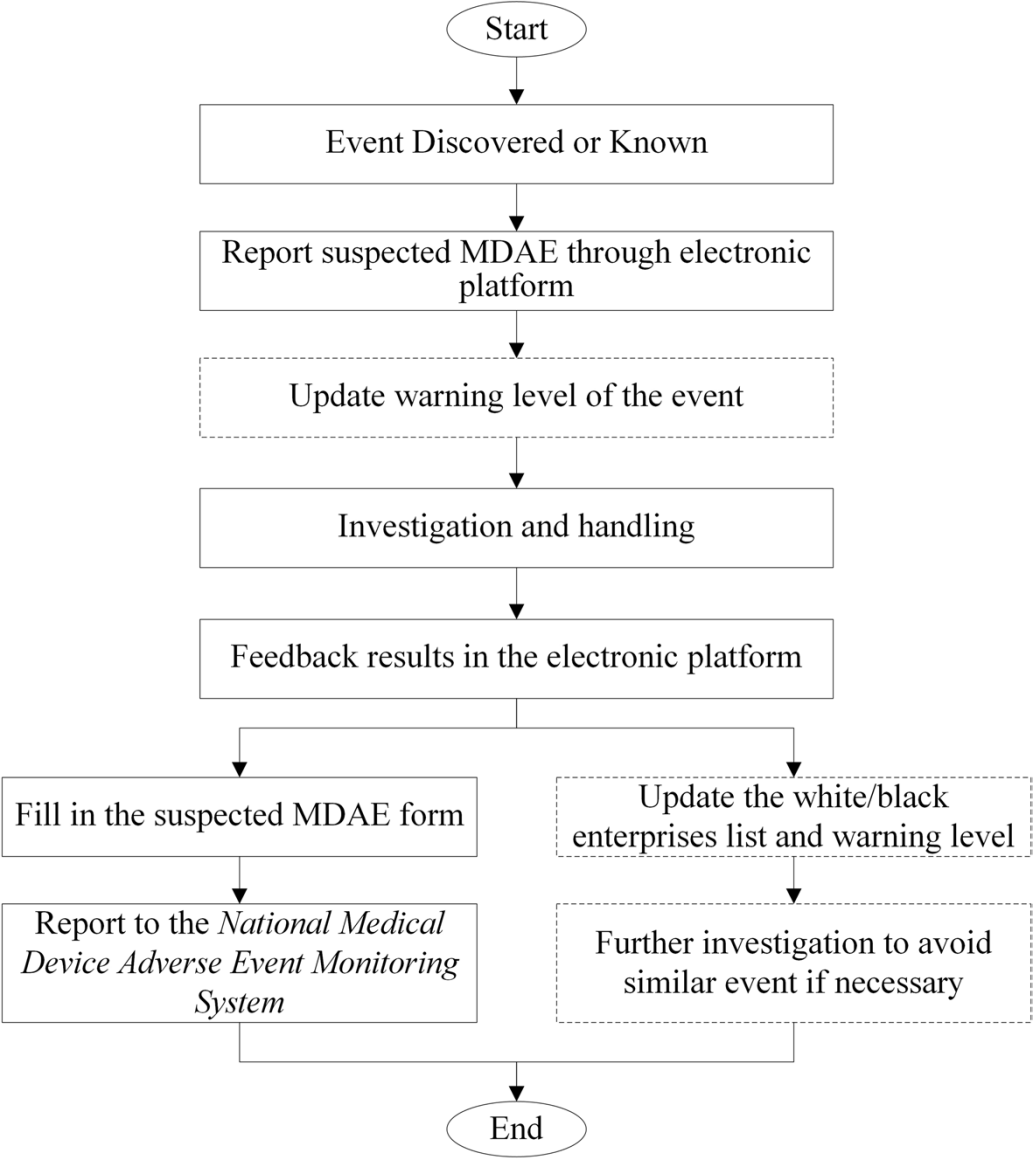
Workflow of medical device adverse events management in a hospital

In accordance with national regulations and experiences, a workflow has been made to standardize the management of MDAE in hospitals, as shown in Fig. 1. MDAE management is the responsibility of a specially assigned employee (the coordinator) from the Department of Clinical Engineering. A customized electronic platform allows all hospital staff to report suspected MDAEs to the coordinator. Upon receiving an MDR, the coordinator must update warning level, followed by an investigation with the purchaser, reporter, supplier and manufacturer of medical devices, as well as other related personnel. Depending on the warning level and the event, the investigation panel and the course of action will differ (see *Responses to Warnings* below). Upon completion of the event, the reporter will receive feedback through the electronic platform. The coordinator then fills out the MDAE reporting form and reports the event to the *National Medical Device Adverse Event Monitoring Information System*; in the meantime, he/she will update the black/white enterprises list and adjust the warning level if necessary. Further investigation will be conducted to prevent a repeat of the incident if necessary.

### Design of a dynamic warning system for MDAE management

**Fig. 2:**
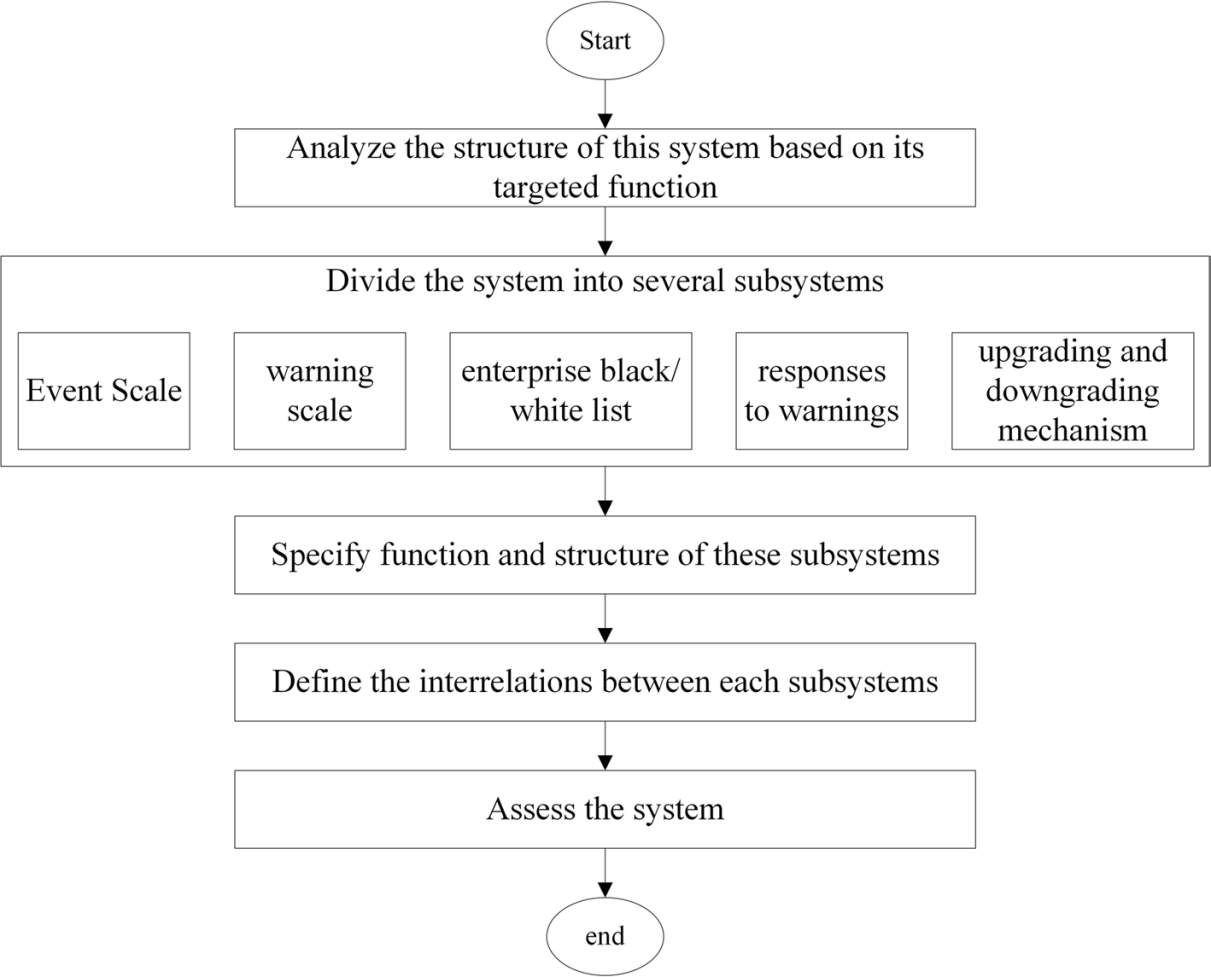
Main steps to design the hospital-based dynamic warning system

According to the above flowchart, warning level of suspected MDAEs is crucial as it determines how to manage the event. A hospital-based dynamic warning system (the system for short below) was then designed by implementing the following five steps (Fig. 2). First, the structure of this system was analyzed based on targeted function. Secondly, this system was divided into five subsystems, including event scale, warning scale, enterprise black/white list, responses to warnings, and upgrading and downgrading mechanism. Thirdly, the function and structure of these subsystems was specified, followed by defining the interrelations between subsystems. Lastly, the function of this system was assessed using historical adverse event data.

#### Event scale setting

In accordance with government regulation [18], events were categorized as General Event, Serious Event, and Extreme Event based on the severity of harm or damage. General Events cause no damage to users, while Extreme Events cause death to a person or cause damage to a group of people simultaneously. As shown in Table 1, severity values range from 0 to 1, and the higher the severity, the more severe the harm or damage. Initially, the values were set and optimized based on MDRs at the FAHZU. The basic principle of value setting is to avoid excessive or insufficient alarm.

**Table 1:** Events scale with its description and corresponding severity

Event Scale	Description	Severity^a^ (0–1)
General Event	No damage occurred; defects of products can possibly be found through pre-use inspection	0.1
Serious Event	Slight damage happened or is likely to occur if the event would happen again. It can be cured via simple treatment	0.4
	Permanent damage or harm can be avoided only through treatment	0.5
	Permanent damage or harm is possibly to happen	0.6
	Permanent damage or harm happened	0.8
	Life-threatening	0.9
Extreme Event	Causing death; or damage occurred simultaneously on a group of people (more than 3 persons)	1

^a^severity of damage or harm caused by related event, the higher means more serious

#### Warning scale setting

Based on risk values, warnings were divided into five levels (white, blue, orange, red and black) (Table 2). The colors used in this study were similar to those used in a typhoon warning system [19]. Risk is the combination of the probability of event occurrence and its related severity [20]. Due to the difficulty of calculating probability, the frequency was substituted for probability in this study. The numerical value of frequency for a single event is one. According to national regulation, medical devices are classified as low-risk, medium-risk, and high-risk levels, so risk value is calculated by summing up the event risk values caused by the same kinds of products with the same brand over the past one month, six months, and one year, respectively (Table 3). Higher risk values indicate that the event needs to be addressed more carefully.

**Table 2:** Warning scale

Warning Level	Risk^*^
White	(0,0.1]
Blue	(0.1,0.3]
Orange	(0.3, 0.5]
Red	(0.5,0.8]
Black	> 0.8

^*^Risk = Frequency * Severity

**Table 3:** Definition of frequency event in this study

risk level of medical device determined by SFDA	frequency defined as the quantity of similar event happening within the past x months
low-risk	one month
medium-risk	six months
high-risk	one year

#### Enterprise black/white list establishment

A Black/White List of Enterprises was established based on risk values to support procurement decision-making. A manufacturer’s risk value is the sum of risk values of events related to the same manufacture. As defined in *Warning Scale* part, the event risk value is adjusted on a daily basis. In the Black/White list, the manufactures are listed in descending order.

#### Responses to warnings

**Fig. 3:**
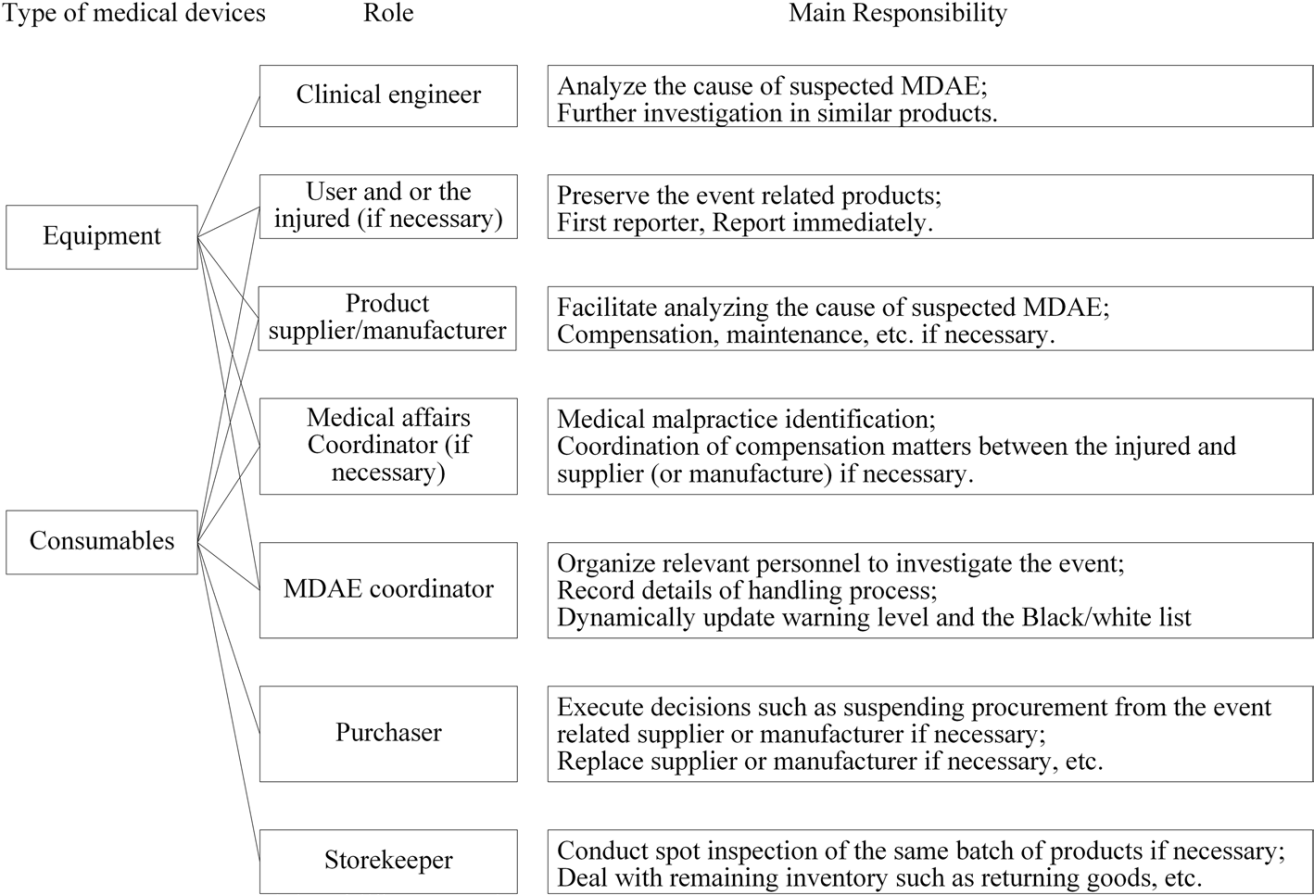
Suspected MDAE related roles and their responsibilities

It is the coordinator’s responsibility to update the warning level immediately following an event. In cases of extreme events, the coordinator should immediately and directly notify the local Market Supervision and Administration Bureau. It is imperative that relevant personnel, including purchasers or clinical engineers, clinical users, suppliers or manufactures, and others (Fig. 3), be organized as soon as possible to investigate the event. There are corresponding responsibilities associated with each role. Based on the investigation result, the warning level and the White/Black List are then adjusted if necessary.

#### Upgrading and downgrading mechanism

Since risk value is the sum of event risk values over the past one, six and twelve months based on the risk level of medical devices and consumables, risk value could be calculated daily. As a result of the dynamic updating of risk values, the warning level and White/Black List are updated in real-time.

### System assessment

In this study, descriptive statistics was used to analyze the data. The MDAE monitoring was initiated in 2010 in the FAHZU by manually filling out forms without mature system. On May 1 in 2011, the first MDAE monitoring system was implemented for suspected adverse events, which has been improved over the past decade. At the end of 2011, an electronic MDAE reporting platform was implemented at the hospital, which has become the main source of MDAE reports. To test the effectiveness of the MDAE monitoring system, the numbers and compound growth rate of MDAE reports obtained through electronic platform, along with annual total MDAE reports from 2010 to 2020, were calculated and compared. Since the data prior to 2013 was not well stored, the number of MDR reported to the *National Adverse Event Monitoring System* from 2013 to 2020 was compared.

By analyzing the risk and warning levels of MDAEs and comparing risk values of manufactures in 2020, the effectiveness and usability of the hospital-based dynamic warning system was then verified. In addition, the causes of the events were analyzed to provide manufactures with suggestions for improving quality. The risk values of events and manufactures were calculated using the aforementioned methods. As described in the Warning Scale Setting section, the risk value of an event is defined as the total of event risk values caused by the same kinds of products with the same brand throughout a certain time period. The risk value of an event is dynamically updated. Based on event risk values associated with each event, warning levels were determined. The risk value of a manufacturer is the sum of the risk values of events associated with the same manufacture.

## Results

**Fig. 4:**
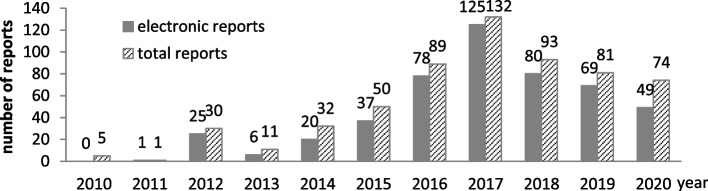
Number of MDRs from 2010 to 2020

Prior to the implementation of the MDAE management system on May 1 in 2011, only five MDRs were reported in 2010. Before the electronic MDAE reporting platform went into use at the end of 2011, only one MDR was reported in 2011. In 2012, the number of reports increased rapidly, reaching 25 electronic reports and 30 reports in total. The number of MDRs increased continuously from 2013 to 2017 and then decreased, indicating a gradual maturity in MDAE management in the hospital (Fig. 4). Each year, more than half of the MDRs were submitted electronically. The compound growth rate from 2013 to 2017 was as high as 113.6%, which from 2017 to 2020 was -26.8%. The total compound growth rate from 2013 to 2020 was 35.0%.

**Fig. 5:**
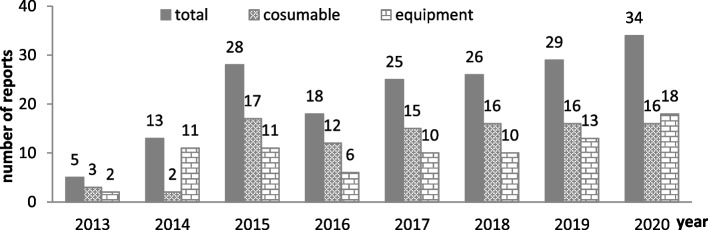
Number of MDRs submitted to the *National Adverse Event Monitoring System* from 2013 to 2020

Suspected MDAEs should be further investigated to distinguish between MDAEs, safety incidents and medical malpractices. The validated MDAEs were then reported to the *National Adverse Event Monitoring System* according to the principle of reporting. Since 2013, the number of MDAEs reported to the *National Adverse Event Monitoring System* went through an upward trend (Fig. 5). The compound growth rate from 2013 to 2020 was 31.5%, indicating an increasing ability of MDAE monitoring.

By the end of 2020, 74 suspected MDAEs were submitted, of which 8 were eliminated due to a lack of product information such as the registration certificate number and manufacturer, or because the event was actually a safety incident. As a result, 66 events were analyzed, including 28 white warnings, 6 blue warnings, 21 orange warnings, 5 red warnings and 6 black warnings. A Black/White list has been created. Among the 34 manufactures on the Black/White list at the end of 2020, the highest risk value was 1.3 (Table 4). Moreover, there were 17 manufactures with risk values greater than 0.1. The risk values of three manufactures exceeded 1, indicating a relatively high risk associated with the product.

**Table 4:** Risk values of part of manufactures at the end of 2020. Names of those manufactures are replaced by number to avoid conflicts

manufacture number	manufacture risk value
1	1.3
2	1.2
3	1.2
4	0.9
5	0.8
6	0.8
7	0.6
8	0.6
9	0.5
10	0.5
11	0.5
12	0.5
13	0.4
14	0.4
15	0.4
16	0.4
17	0.2

After investigation, probable causes of adverse events could be categorized as poor quality, design defect, and improper operation (Table 5). Fifty-nine suspected MDAEs were caused by poor quality of products, indicating that these manufactures should improve their products’ quality. Design defects was the second leading cause of MDAEs, indicating that manufactures should improve design of products for better clinical use. Improper operation on medical device was also the cause of adverse events, indicating that more manipulation training should be provided to users of these medical devices.

**Table 5:** Causes of adverse events reported in 2020

causes of event	kinds of devices	number of events
**poor quality**		**59**
	medical device	7
	consumables	52
**design defect**		**6**
	medical device	6
	consumables	0
**improper operation**		**1**
	medical device	0
	consumables	1

## Discussions and conclusions

This study illustrated a MDAE monitoring system for suspected adverse events and a hospital-based dynamic warning system for MDAE management. The systems were implemented in the FAHZU. The compound annual growth rates of MDAEs reports and MDRs submitted to the *National Medical Device Adverse Event Monitoring Information System* from 2013 to 2020 were 35.0% and 31.5%, respectively, indicating an increasing ability of MDAE monitoring. Results showed that a hospital-wide standard for the management of suspected MDAEs with full participant, timely response, and efficient feedback was feasible. This system improved the monitoring level of suspected MDAEs, which improved early detection, early warning and early intervention of risks associated with medical device. In addition to providing guidance to purchasers of medical devices in hospitals, the enterprise black/white list can encourage manufactures of medical devices to improve the quality of their products. As a result of this system, MDAEs can be processed effectively, and hospitals can monitor MDAEs in real-time. It is possible to provide recommendations for medical institutions in regards to the development of a MDAE monitoring system or the improvement of the existing one in terms of early detection, early warning and early intervention of risk related to medical devices. Additionally, this system may provide useful information for procurement decision-making as well as further promote quality and safety management in hospitals to some extent.

MDAE regulatory system was first established in 2007 in the FAHZU. Following the constant changes in relevant national regulations and accumulation of experience, MDAE regulatory system and workflow have been continuously optimized and improved, resulting in an increasing number of reports being submitted to the *National Medical Device Adverse Event Monitoring Information System*. The increase in the number of MDRs likely results from improved monitoring system and increased awareness of MDAE reporting. Similar results have been reported in studies around the world [21–23]. There is also evidence that incentives can improve the quantity and quality of MDRs in other studies [24, 25]. In the future, effective incentives could be introduced into this monitoring system and further research could be conducted.

An important objective of MDAE monitoring is to provide early detection, early warning and early intervention. There are a few studies related to MDAE prediction [22, 26], but few studies about MDAE warning have been conducted. The dynamic warning system that has been designed in this study is innovative. As of today, early warning system (EWS) of nature disasters such as extreme weather, avalanches, and earthquakes are remarkably effective in preventing damage through similar real-time monitoring [27]. The EWS provided better suggestion for decision-making by combining impact and probability, which are also considered in this dynamic warning system [28]. However, the dynamic warning system is a retrospective analysis, and since MDAE occurrence is highly unpredictable, the probability is replaced by occurrence per month, six months, and year. The dynamic warning system uses color for warning because it is more comprehensible than charts and reports, which are often utilized in hazard warning labels [29, 30]. The colors selected in this study mimicked those used in a typhoon warning system. Moreover, a white level was added in place of the yellow level. Different colors convey a different level of urgency to people.

After the deployment of the monitoring system, especially after the introduction of the electronic reporting platform, the number of MDAE reports increased dramatically, showing an ongoing improvement in MDAE management. In 2013 and 2018, however, the number of MDAE reports decreased. This was due to the hospital’s proposal to integrate the electronic MDAE reporting platform into another medical quality monitoring platform in 2013 and again in 2017. Ultimately, the policy was withdrawn, but a new hospital management information system was prepared for redevelopment in 2018 and began development in 2019. Training and publicity of the reporting system were affected by changes in hospital policy. Growth in report number is the leading indicator of the MDAE management level. There were, however, limitations in the assessment methods used in this study. It is possible to investigate the level of awareness of MDAE reporting among nurses, clinicians, clinical engineers, etc., through questionnaires and interviews. The quality of MDAE reports can also be further compared using an evaluation scale.

However, ongoing optimization is necessary in order to improve the overall functionality and effectiveness of the system, especially in terms of alarm management and work efficiency. On the one hand, alarm fatigue was detrimental to clinicians [31], as well as to the coordinator of MDAE management; thus, this system should be further optimized in aspects such as risk value setting through application, aiming to achieve more effective early warning, i.e., to avoid excessive warning and untimely warning. On the other hand, electronic submission and management is an effective means to help process, review, and archive MDRs [32] and to improve operation efficiency. Therefore, a computerized MDAE alarm system based on these findings should be developed in the future. The computerized MDAE alarm system may be further developed by using big data analytics and artificial intelligence, in order to reveal deficiencies in medical devices in a timely fashion. The findings of deficient medical devices not only can provide timely evidence in support of registration and recall, but can also provide novel insights for assessing improvements in standards and further promoting the optimization of medical devices [33, 34], especially with the increasing application of artificial intelligence in medical devices [35]. MDAEs prediction is important for post-market surveillance and appropriate public health interventions [26]; therefore, as medical Internet of things and artificial intelligence technologies progress, more extensive medical data could be used for the detection and warning of MDAEs. Due to the rapid growth of the medical device market and the increasing complexity of medical devices, the number of MDAEs keeps increasing, placing a greater strain on governmental post-market supervision. According to the National Medical Device Adverse Event Monitoring Information System, 650,695 adverse events were reported in 2021, an increase of 21.39% from 2020 [36]. In light of the large number of MDRs that are occurring nationally and in the process of growing, it may be more appropriate to have a national MDAE alarm system for pre-market testing and post-market supervision of medical devices.

## Data Availability

The datasets used and/or analyzed during the current study available from the corresponding author on reasonable request.

## Abbreviations

FDA: US food and drug administration
MDAE: Medical device adverse event
MDR: MDAE report
